# Longitudinal assessment of urinary ALCAM, HPX, and PRDX6 in Korean patients with systemic lupus erythematosus: implications for disease activity monitoring and treatment response

**DOI:** 10.3389/fimmu.2024.1369385

**Published:** 2024-06-10

**Authors:** Ji-Won Kim, Wook-Young Baek, Ju-Yang Jung, Hyoun-Ah Kim, Sang-Won Lee, Chang-Hee Suh

**Affiliations:** ^1^ Department of Rheumatology, Ajou University School of Medicine, Suwon, Republic of Korea; ^2^ Department of Molecular Science and Technology, Ajou University, Suwon, Republic of Korea

**Keywords:** urine biomarker, systemic lupus erythematosus, ALCAM, HPX, PRDX6

## Abstract

**Introduction:**

This study aimed to demonstrate the potential of activated leukocyte cell adhesion molecule (ALCAM), hemopexin (HPX), and peroxiredoxin 6 (PRDX6) as urine biomarkers for systemic lupus erythematosus (SLE).

**Methods:**

Urine samples were collected from 138 Korean patients with SLE from the Ajou Lupus Cohort and 39 healthy controls (HC). The concentrations of urine biomarkers were analyzed using enzyme-linked immunosorbent assay kits specific for ALCAM, HPX, and PRDX6, respectively. Receiver operating characteristic (ROC) curve analysis was performed to evaluate the diagnostic utility, and Pearson’s correlation analysis was conducted to assess the relationships between the disease activity and urine biomarkers.

**Results:**

Patients with SLE and patients with lupus nephritis (LN) showed significantly elevated ALCAM, HPX, and PRDX6 levels compared with HCs. ALCAM, HPX, and PRDX6 showed significant diagnostic values, especially for lupus nephritis (LN), with areas under the receiver operating characteristic curve for LN was 0.850 for ALCAM (95% CI, 0.778–0.921), 0.781 for HPX (95% CI, 0.695–0.867), and 0.714 for PRDX6 (95% CI, 0.617–0.812). Correlation analysis revealed that all proteins were significantly associated with anti-double stranded DNA antibody (ALCAM, r = 0.350, p < 0.001; HPX, r = 0.346, p < 0.001; PRDX6, r = 0.191, p = 0.026) and SLEDAI (ALCAM, r = 0.526, p < 0.001; HPX, r = 0.479, p < 0.001; PRDX6, r = 0.262, p = 0.002). Results from the follow-up of the three biomarker levels in these patients revealed a significant decrease, showing a positive correlation with changes in SLEDAI-2k scores (ALCAM, r = 0.502, p < 0.001; HPX, r = 0.475, p < 0.001; PRDX6, r = 0.245, p = 0.026), indicating their potential as indicators for tracking disease activity.

**Discussions:**

Urinary ALCAM, HPX, and PRDX6 levels have diagnostic value and reflect disease activity in Korean patients with SLE, emphasizing their potential for non-invasive monitoring and treatment response evaluation.

## 1 Introduction

Systemic lupus erythematosus (SLE) is a systemic autoimmune disease characterized by the presence of autoantibodies and immune complexes that damage various organs, tissues, and cells (1, 2). The development of SLE is attributed to a combination of factors such as environmental triggers, genetic predisposition, hormonal influences, and the presence of autoantibodies. These factors lead to a malfunction of the immune system, resulting in an inability to differentiate between self and non-self, and ultimately leading to the immune system attacking healthy cells. Depending on the tissues affected by these attacks, various symptoms can occur, including skin rashes, photosensitivity, arthritis, nephritis, stomatitis, cytopenia, and vasculitis (3, 4).

Diagnosing SLE can be challenging because of the diverse and often nonspecific symptoms that which can resemble those of other medical conditions. The diagnostic process for SLE involves a comprehensive approach that relies on a combination of clinical evaluation, laboratory tests, and the exclusion of other possible diagnoses (5). In SLE patients with suspected lupus nephritis (LN), in addition to the various tests mentioned earlier, renal biopsy using invasive needle procedures is essential for diagnosis. Furthermore, as LN carries a high risk of progression to end-stage renal disease and can necessitate renal replacement therapy in later stages of life and repeat renal biopsies may be required during the course of treatment (6). Therefore, continuous efforts have been made in the field of SLE to discover biomarkers that can enable timely diagnosis, track disease activity, and assess treatment efficacy. Due to its less invasive nature and physical proximity to the active sites of renal disease, urine is considered a promising biomarker for monitoring LN activity (7).

In a recent study, researchers evaluated LN by screening urine samples from patients with active LN and analyzing 1,129 proteins using an aptamer-based platform. This study successfully identified specific urine proteins, namely activated leukocyte cell adhesion molecule (ALCAM), hemopexin (HPX), and peroxiredoxin 6 (PRDX6), which were found to be effective in identifying the disease (8, 9). ALCAM, also known as cluster of differentiation 166 (CD166), is a cell surface protein involved in inflammation and immune responses (10). HPX is a protein that regulates heme biology to maintain iron balance. It is primarily expressed in the liver and acts as an acute-phase reactant during inflammation (11). PRDX6 is an enzyme that has dual functions of glutathione peroxidase and phospholipase A2 and plays a significant role in pathological processes (12).

In this study, we aimed to assess the potential diagnostic capabilities of these urine biomarkers for SLE and LN in Korean patients and analyze their associations with clinical symptoms and disease activity. Furthermore, we aimed to evaluate changes in urine biomarkers based on treatment outcomes.

## 2 Materials and methods

### 2.1 Study population and clinical assessments

This single-center, retrospective study was approved by the Ajou University Hospital Institutional Review Board (AJIRB-OBS-2015–423). The Ajou lupus cohort included individuals aged 18 and above with a confirmed diagnosis of SLE based on either the 2012 Systemic Lupus International Collaborating Clinics or the 2019 European League against Rheumatism/American College of Rheumatology criteria (5, 13). The exclusion criteria included patients with other autoimmune diseases such as Sjogren’s syndrome, rheumatoid arthritis, and systemic sclerosis. Participants who consented to urine sample collection underwent a comprehensive retrospective review of their medical records, which included an examination of their demographic, laboratory, and clinical information.

During outpatient consultations, the patients were questioned about the presence of oral ulcers, skin rashes, arthritis, alopecia, and fever. Laboratory tests included complete blood count, erythrocyte sedimentation rate (ESR), renal function, urine protein-to-creatinine ratio (UPCR), complement 3 (C3), complement 4 (C4), and autoantibodies, including antinuclear antibody (ANA) and anti-double-stranded DNA (anti-dsDNA) antibody. Anti-dsDNA antibodies were assessed using the Anti-dsDNA kit (Trinity Biotech, Bray, Ireland), with values exceeding 7 IU/ml considered abnormal. C3 and C4 levels were assessed using Cobas (Roche Diagnostics, Basel, Switzerland), with a normal range of 90–180 mg/dl for C3 and 10–40 mg/dl for C4. The Systemic Lupus Erythematosus Disease Activity Index 2000 (SLEDAI-2k) was calculated based on patient records and laboratory test results.

### 2.2 Measurement of urine biomarkers

We collected urine samples from healthy controls (HC) (n=39), patients with SLE (n=138), of which 71 had active LN at the time of urine collection and the other 67 had active non-renal SLE. We immediately stored at -80°C after sample collection. The concentration of biomarker levels in mouse urine was analyzed using enzyme-linked immunosorbent assay (ELISA) kits specific for ALCAM (Catalog # DY656; R&D Systems, Minneapolis, MN, USA), HPX (Catalog # ab108860; Abcam Inc, Toronto, ON, Canada) and PRDX 6 (Catalog # MBS067069; Mybiosource, San Diego, CA, USA), according to the manufacturer’s protocols. The dilutions were selected to ensure that all biomarker concentrations were within the optimal range for the assays.

We analyzed longitudinal changes in the urine biomarkers ALCAM, HPX, and PRDX6 in patients with SLE during the treatment. Biomarker measurements at both the initial and follow–up stages were performed using the same methodology.

### 2.3 Data analysis

Statistical analyses were conducted using SPSS software (version 25.0; IBM Corporation, Armonk, NY, USA). Results are presented as mean ± SD, with statistical significance set at p <0.05. Baseline population differences were assessed for continuous variables using either the Student’s t–test or Mann–Whitney U test, whereas categorical variables were analyzed using the chi-square test or Fisher’s exact test. The utility of urine biomarker levels as diagnostic markers to distinguish patients with SLE from HCs was established by analyzing the area under the Receiver Operating Characteristic (ROC) curve (AUC). To evaluate the positivity rate of the biomarker combination, we conducted the upper limit of the 95% confidence interval. Additionally, we constructed a logistic regression model to evaluate the diagnostic performance of the biomarker combination. Using this model, we assessed the AUC, sensitivity, specificity, positive predictive value (PPV), and negative predictive value (NPV). Confusion matrix and Youden Index were employed to evaluate the model’s performance (14).

## 3 Results

### 3.1 Clinical and demographic features

The demographic and disease characteristics of the study participants, including individuals with SLE and HCs, are summarized in **Table 1**. The classification of renal biopsy in patients with LN is presented in **Supplementary Table 1**. Statistical analyses revealed no significant differences in age or female-to-male ratio between patients with SLE and HCs. The mean disease duration was significantly longer in SLE patients with LN compared to those without LN, with values of 123.1 ± 19.2 months and 93.2 ± 81 months, respectively. No significant differences were observed in the concurrent clinical symptoms. Regarding laboratory tests, a significantly higher proportion of patients with LN exhibited reduced complement levels and positive anti-dsDNA antibody than patients with SLE without LN. Additionally, mean proteinuria and SLEDAI-2k scores were significantly higher in the LN group. In terms of SLE treatment medications, non-steroidal anti-inflammatory drug use was more prevalent in patients SLE without LN. Conversely, patients with SLE and LN demonstrated higher utilization rates of glucocorticoids, mycophenolate mofetil, calcineurin inhibitors, and angiotensin-converting enzyme inhibitors/angiotensin receptor blockers.

**Table 1:** Demographic and clinical characteristics of patients with systemic lupus erythematosus and healthy controls.

Variable	SLE without LN (N = 67)	SLE with LN (N = 71)	Healthy controls (N = 39)	P-value
Age, years (mean ± SD)	42.4 ± 11.4	39.1 ± 11.5	40.8 ± 8.91	0.183
Female, no. (%)	66 (98.5)	67 (94.4)	37 (94.9)	0.424
Disease duration, months	93.2 ± 81	123.1 ± 19.2		**0.03**
Alcohol, no. (%)	16 (23.9)	19 (26.8)		0.698
Smoking, no. (%)	5 (7.5)	10 (14.1)		0.212
Clinical manifestations				0.210
Fever, no. (%)	1 (1.5)	5 (6.9)		
Mucocutaneous, no. (%)	29(43.3)	27 (38.0)		0.530
Arthritis, no. (%)	20 (29.9)	22 (31.0)		0.885
Myositis, no. (%)	0 (0)	2 (2.8)		0.497
Serositis, no. (%)	0 (0)	1 (1.4)		>0.999
Hematologic, no. (%)	21 (31.3)	23 (32.4)		0.895
Central nervous system, no. (%)	0 (0)	2 (2.8)		0.497
Laboratory finding				
Leukocyte,/μL	4,709.0 ± 1,970.0	5,156.3 ± 2,509.5		0.245
Lymphocyte,/μL	1,518.4 ± 657.0	1,369.3 ± 733.2		0.211
Platelets, ×10^3^/μL	208.8 ± 75.9	216.7 ± 64.7		0.507
ESR, mm/h	13.5 ± 13.0	17.1 ± 14.8		0.147
Complement 3, mg/dL	92.6 ± 20.3	72.6 ± 24.0		**<0.001**
Complement 4, mg/dL	19.5 ± 7.9	14.7 ± 9.5		**0.002**
Anti-ds DNA (IU/mL)	33.2 ± 126.2	72.9 ± 133.3		0.074
Immunologic finding				
ANA positivity, no. (%)	64 (95.5)	68 (95.8)		>0.999
Anti-ds DNA Ab positivity, no. (%)	17 (25.4)	44 (62.0)		**<0.001**
Anti-Sm Ab positivity, no. (%)	9 (13.4)	17 (23.9)		0.097
Anti-PL Ab positivity, no. (%)	20 (29.9)	21 (29.6)		0.972
Low complements (C3 < 90mg/dL or C4 < 10mg/dL), no. (%)	31 (46.3)	55 (77.5)		**<0.001**
Urinalysis				
Proteinuria (mg/day)	0.13 ± 0.17	0.87 ± 1.34		**0.001**
Proteinuria >0.5 g/day, no. (%)	3 (4.5)	34 (47.9)		**<0.001**
SLEDAI-2k	3.69 ± 3.38	10.3 ± 7.01		**<0.001**
Treatment				
Hydroxychloroquine, no. (%)	66 (98.5)	71 (100)		0.486
NSAID, no. (%)	23 (34.3)	9 (12.7)		**0.003**
Glucocorticoids, no. (%)	39 (58.2)	63 (88.7)		**<0.001**
GC dose, mg/day, median, IQR (prednisolone-equivalent)	2.5 (0.625–5.0)	5.0 (2.5–7.5)		0.495
Immunosuppressants no. (%)				
Azathioprine, no. (%)	3 (4.5)	10 (14.1)		0.053
Mycophenolate mofetil, no. (%)	0 (0)	30 (42.3)		**<0.001**
Cyclophosphamide, no. (%)	1 (1.5)	2 (2.8)		>0.999
Calcineurin inhibitor, no. (%)	7 (10.4)	27 (38.0)		**<0.001**
Mizoribine, no. (%)	7 (10.4)	7 (9.9)		0.909
ACE inhibitor or ARB, no. (%)	1 (1.5)	41 (57.7)		**<0.001**
Vitamin D, no. (%)	53 (79.1)	52 (73.2)		0.420

SLE, systemic lupus erythematosus; LN, lupus nephritis; ESR, erythrocyte sedimentation rate; ANA, anti-nuclear antibody; ds-DNA, double-strand deoxyribonucleic acid; Ab, antibody; Sm, Smith; PL, phospholipid; C3, complement 3; C4, complement 4; SLEDAI-2k, SLE disease activity index 2000; NSAID, nonsteroidal anti-inflammatory drugs; GC, glucocorticoid; IQR, interquartile range; ACE, angiotensin-converting enzyme; ARB, angiotensin receptor blocker.

Bold values indicate significant P-values.

### 3.2 Urine levels of ALCAM, HPX, and PRDX6 in patients with SLE and HCs

**Figure 1:**
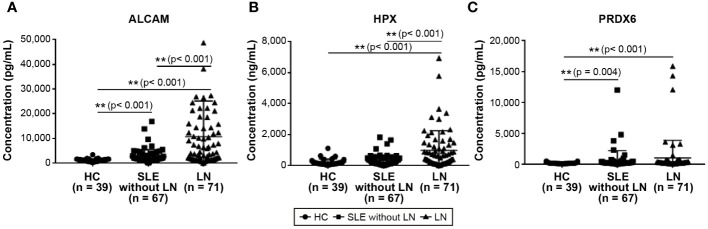
Comparison of urine biomarkers among patients with SLE, LN, and healthy controls. **(A)** Urine level of ALCAM. **(B)** Urine level of HPX. **(C)** Urine level of PRDX6. Statistical analyses were conducted using the Mann–Whitney U test. SLE, systemic lupus erythematosus; LN, lupus nephritis; ALCAM, activated leukocyte cell adhesion molecule; HPX, hemopexin; PRDX, peroxiredoxin.

**Figure 1** depicts the concentrations of urinary ALCAM, HPX, and PRDX6 in patients with SLE (without LN), LN, and HCs. In **Figure 1A**, the comparative graph for ALCAM reveals a statistically significant elevation in SLE without LN (2,601.4 ± 2,826.4 pg/ml) and LN patients (10,685.3 ± 14,488.5 pg/ml) compared to HCs (1,192.6 ± 577.0 pg/ml). **Figure 1B**, illustrating HPX, shows a statistically significant increase in LN patients (968.8 ± 1,275.9 ng/ml) compared to both HC (202.0 ± 211.2 ng/ml) and SLE patients without LN (316.4 ± 353.0 ng/ml) (p<0.001). In **Figure 1C**, the comparison of PRDX6 concentrations shows a consistently lower level in HC (174.5 ± 97.9 pg/ml) compared to SLE (602.9 ± 1610.5 pg/ml; p = 0.004) and LN (1004.7 ± 2863.0 pg/ml; p<0.001) patients. Both the HC vs. SLE and HC vs. LN comparisons demonstrated statistically significant differences.

### 3.3 ROC curves for the diagnosis of SLE using urine ALCAM, HPX, and PRDX6

**Figure 2:**
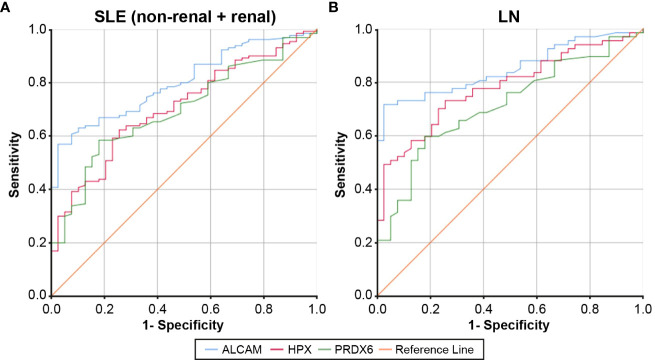
ROC curves for urine ALCAM, HPX, and PRDX in the diagnosis of SLE and LN compared to healthy controls. **(A)** For SLE (non-renal + renal) diagnosis, the AUC was 0.801 for the urine ALCAM (95% CI, 0.734 – 0.868), 0.707 for the urine hemopexin (95% CI, 0.622 – 0.792), and 0.697 for the urine PRDX (95% CI, 0.612 – 0.782). **(B)** For LN diagnosis, the AUC was 0.850 for the urine ALCAM (95% CI, 0.778 – 0.921), 0.781 for the urine hemopexin (95% CI, 0.695 – 0.867), and 0.714 for the urine PRDX (95% CI, 0.617 – 0.812). ROC, Receiver operating characteristic; SLE, systemic lupus erythematosus; LN, lupus nephritis; ALCAM, activated leukocyte cell adhesion molecule; HPX, hemopexin; PRDX, peroxiredoxin; AUC, area under the receiver operating characteristic curve; CI, confidence interval.

**Figure 2** displays the ROC curves for the urine levels of ALCAM, HPX, and PRDX6 in discriminating between SLE with and without LN and LN specifically. The optimal cut-off values for SLE diagnostic markers were determined as 1,898.7 pg/ml for ALCAM, 209.3 ng/ml for HPX, and 0.20 pg/ml for PRDX6. The AUC values for ALCAM, HPX, and PRDX6 were 0.801 (95% CI, 0.734 – 0.868), 0.707 (95% CI, 0.622 – 0.792), and 0.697 (95% CI, 0.612–0.782), respectively (**Supplementary Table 2**). The optimal cut-off values for LN diagnostic markers were determined as 1,935 pg/ml for ALCAM, 212.5 ng/ml for HPX, and 59.7 pg/ml for PRDX6. The AUC values for ALCAM, HPX, and PRDX6 were 0.850 (95% CI, 0.778 – 0.921), 0.781 (95% CI, 0.695 – 0.867), and 0.714 (95% CI, 0.617–0.812), respectively (**Supplementary Table 3**). **Supplementary Tables 2**, **3** provide the diagnostic performance characteristics, including sensitivity, specificity, positive predictive value, and negative predictive value, of the biomarkers used in diagnosing SLE and LN. Among the three biomarkers, ALCAM demonstrated the highest specificity and positive predictive value in both the SLE and LN cohorts. The specificity of ALCAM in both SLE and LN was 97.4%, with positive predictive rates of 98.7% in SLE and 98% in LN, indicating a remarkably robust diagnostic performance. Additionally, the diagnostic performance of each biomarker combination was presented in **Supplementary Tables 4**, **5**. When all three biomarkers were utilized, the AUC for diagnosing SLE reached 0.858, and for diagnosing LN, it reached 0.888, demonstrating very good diagnostic capabilities.

### 3.4 Correlations of urine biomarker with SLE disease activity and clinical manifestations

We conducted a correlation analysis to explore the associations between urinary biomarkers (ALCAM, HPX, PRDX6) and hematological markers related to SLE disease activity (**Table 2**). Urinary ALCAM was positively correlated with leukocyte count (r = 0.251, p = 0.003), anti-dsDNA antibody levels (r = 0.350, p <0.001), UPCR (r = 0.515, p <0.001), and SLEDAI-2k (r = 0.525, p <0.001), and negatively correlated with hemoglobin (r = -0.332, p <0.001) and C3 (r = -0.226, p = 0.008). Similarly, HPX demonstrated generally concordant results, showing positive correlations with leukocyte count (r = 0.245, p = 0.004), anti-dsDNA antibody levels (r = 0.346, p <0.001), UPCR (r = 0.369, p <0.001), and SLEDAI-2k (r = 0.479, p <0.001), and negative correlations with hemoglobin (r = -0.257, p = 0.003) and C3 (r = -0.173, p = 0.046). PRDX6 was positively correlated with anti-dsDNA antibody (r = 0.191, p = 0.026) and SLEDAI-2k (r = 0.262, p = 0.002).

**Table 2:** Correlation between urine biomarkers and disease activity markers in patients with SLE.

Disease activity markers	Correlation coefficient, *r* (p-value)		
	ALCAM	HPX	PRDX6
Leukocyte	0.251 (**0.003**)	0.245 (**0.004**)	0.022 (0.797)
Lymphocyte	0.065 (0.451)	0.074 (0.398)	0.010 (0.912)
Hemoglobin	-0.332 (**<0.001**)	-0.257 (**0.003**)	-0.155 (0.071)
Platelet	0.101 (0.242)	0.068 (0.432)	-0.010 (0.907)
ESR	0.165 (0.057)	0.011 (0.900)	0.068 (0.434)
Complement 3	-0.226 (**0.008**)	-0.173 (**0.046**)	-0.086 (0.318)
Complement 4	-0.125 (0.147)	-0.096 (0.270)	-0.045 (0.606)
Anti-ds DNA Ab	0.350 (**<0.001**)	0.346 (**<0.001**)	0.191 (**0.026**)
UPCR	0.515 (**<0.001**)	0.369 (**<0.001**)	0.081 (0.411)
SLEDAI-2k	0.526 (**<0.001**)	0.479 (**<0.001**)	0.262 (**0.002**)

SLE, systemic lupus erythematosus; ALCAM, activated leukocyte cell adhesion molecule; HPX, hemopexin; PRDX, peroxiredoxin; ESR, erythrocyte sedimentation rate; ds-DNA, double-strand deoxyribonucleic acid; Ab, antibody; UPCR, urine protein creatinine ratio; SLEDAI-2k, SLE disease activity index 2000. Bold values indicate significant P value.

We also analyzed the correlation between clinical symptoms of SLE and urinary biomarkers, and the results are presented in **Table 3**. Additionally, we analyzed the correlation between clinical symptoms of SLE and urinary biomarkers, and the results are presented in **Table 3**. PRDX6 did not significantly correlate with clinical symptoms. In contrast, ALCAM and HPX are associated with nephritis and central nervous system (CNS) involvement. Both biomarkers were significantly elevated in patients with nephritis and CNS involvement (p < 0.001 for both).

**Table 3:** Comparison of ALCAM, HPX, and PRDX according to clinical manifestations in patients with SLE.

Manifestations	ALCAM	P-value	HPX	P-value	PRDX6	P-value
Fever						
(+) = 6	10,428.0 ± 9,025.8	0.433	724.1 ± 416.6	0.893	0.40 ± 0.20	0.677
(−) = 132	6,694.8 ± 11,444.1		662.0 ± 1,019.8		0.81 ± 2.40	
Oral ulcer						
(+) = 15	7,666.6 ± 8,424.2	0.771	565.9 ± 658.8	0.700	1.13 ± 3.03	0.561
(−) = 123	6,759.5 ± 11,682.4		675.8 ± 1,037.3		0.75 ± 2.26	
Malar rash						
(+) = 8	3,756.1 ± 2,856.7	0.460	630.6 ± 597.4	0.928	1.35 ± 1.87	0.553
(−) = 130	7,027.9 ± 11,612.9		666.2 ± 1,022.3		0.77 ± 2.37	
Arthritis						
(+) = 42	6,957.8 ± 8,790.7	0.947	803.8 ± 918.0	0.295	0.84 ± 2.30	0.894
(−) = 96	6,817.1 ± 12,329.9		605.0 ± 1,036.2		0.78 ± 2.37	
Myositis						
(+) = 2	11,684.3 ± 14,872.4	0.547	1,702.5 ± 2,376.6	0.141	0.32 ± 0.33	0.773
(−) = 136	6,787.5 ± 11,343.4		648.6 ± 980.7		0.80 ± 2.36	
Alopecia						
(+) = 27	10,184.8 ± 13,084.0	0.089	920.4 ± 930.7	0.148	1.50 ± 3.94	0.264
(−) = 111	6,035.8 ± 10,783.5		602.7 ± 1,014.2		0.62 ± 1.72	
Nephritis						
(+) = 71	10,685.3 ± 14,488.5	**<0.001**	968.8 ± 1,275.9	**<0.001**	0.95 ± 2.85	0.379
(−) = 67	2,601.4 ± 2,826.4		316.4 ± 353.0		0.60 ± 1.61	
Serositis						
(+) = 1	3,888.35	0.794	295.0	0.713	0.17	0.789
(−) = 137	6,881.5 ± 11,386.4		667.1 ± 1,006.4		0.80 ± 2.35	
CNS involvement						
(+) = 2	23,371.9 ± 1,656.3	**0.038**	3,383.0 ± 4,215.1	**0.006**	0.43 ± 0.17	0.825
(−) = 0	6,613.1 ± 11,247.7		643.9 ± 978.5		0.80 ± 2.36	

ALCAM, activated leukocyte cell adhesion molecule; HPX, hemopexin; PRDX, peroxiredoxin; SLE, systemic lupus erythematosus; CNS, central nervous system. Bold values indicate significant P value.

“(+)” denotes patients with symptoms, while “(−)” denotes patients without symptoms.

### 3.5 Association between follow-up levels of urinary biomarkers and SLE disease activity

**Figure 3:**
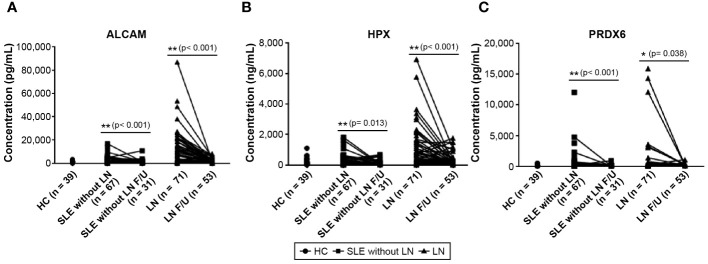
Changes in urine biomarker levels in follow-up observations of patients with SLE without LN and patients with LN. **(A)** Urine level of ALCAM. **(B)** Urine level of HPX. **(C)** Urine level of PRDX6. Statistical analyses were conducted using the Mann–Whitney U test. SLE, systemic lupus erythematosus; LN, lupus nephritis; ALCAM, activated leukocyte cell adhesion molecule; HPX, hemopexin; PRDX, peroxiredoxin.

**Figure 4:**
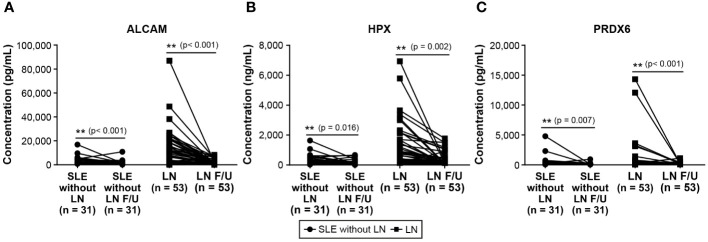
Changes in urine biomarker Levels of patients with SLE without LN and patients with LN only follow-up. **(A)** Urine level of ALCAM. **(B)** Urine level of HPX. **(C)** Urine level of PRDX6. Statistical analyses were conducted using the Mann–Whitney U test. SLE, systemic lupus erythematosus; LN, lupus nephritis; ALCAM, activated leukocyte cell adhesion molecule; HPX, hemopexin; PRDX, peroxiredoxin.

We conducted follow-up assessments of these three biomarkers in patients with SLE. Excluding patients without available follow-up data, we analyzed the follow-up test data of 31 of 67 patients with SLE without LN and 53 of 71 patients with LN. The results revealed a significant decrease in the levels of ALCAM, HPX, and PRDX6 in follow-up tests (**Figure 3**). In the follow-up ALCAM levels, the mean values decreased from 2,601.4 pg/ml to 1,828.3 pg/ml in SLE without LN (p < 0.001) and from 10,685.3 pg/ml to 2,042.6 pg/ml in LN (p < 0.001). For follow-up HPX levels, the mean values decreased from 316.4 ng/ml to 277.5 ng/ml in SLE without LN (p = 0.013) and from 968.8 ng/ml to 337.1 ng/ml in LN (p < 0.001). In follow-up PRDX6 levels, the mean values decreased from 602.9 pg/ml to 162.8 pg/ml in SLE without LN (p = 0.013) and from 1004.7 pg/ml to 224.6 pg/ml in LN (p < 0.001). When comparing only patients who underwent follow-up examinations, there was a definite difference between pre- and post-follow-up values, as illustrated in **Figure 4**. In addition, the longitudinal data for urine ALCAM, HPX, and PRDX6 normalized for urine creatinine were evaluated in each patient who underwent follow-up examinations. In the follow-up ALCAM/creatinine, the mean values decreased from 3,302.8 pg/mg to 1,250.9 pg/mg in SLE without LN (p = 0.002) and from 12,891.6 pg/mg to 2,232.1 pg/mg in LN (p < 0.001). For follow-up HPX/creatinine, the mean values decreased from 339,491.6 pg/mg to 130,036.5 ng/ml in SLE without LN (p = 0.012) and from 1,220,609.7 pg/mg to 342,268.9 pg/mg in LN (p = 0.001). In follow-up PRDX6/creatinine, the mean values decreased from 380.7 pg/ml to 169.4 pg/ml in SLE without LN (p = 0.073) and from 1,317.7 pg/mg to 260.3 pg/mg in LN (p = 0.01). The levels of ALCAM, HPX, and PRDX6 normalized to creatinine remained similar to the original data, as well as their statistical significance (**Supplementary Figure 2**).

**Figure 5:**
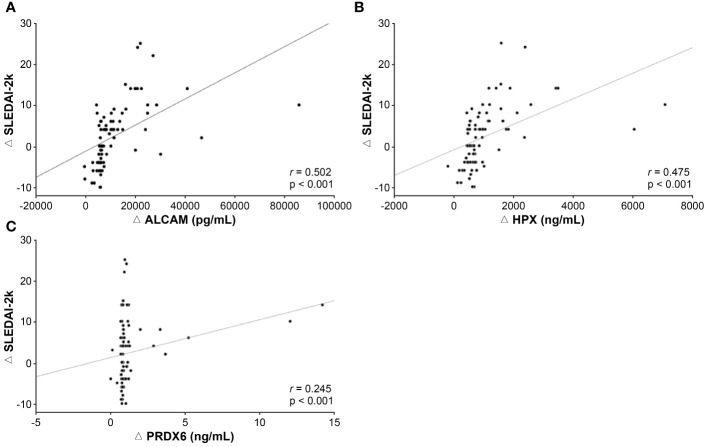
Association between changes in biomarker levels and disease activity during follow-up examinations. **(A)** Correlation between ΔALCAM and ΔSLEDAI-2k. **(B)** Correlation between HPX and SLEDAI-2k. **(C)** Correlation between Δ PRDX6 and ΔSLEDAI-2k. SLEDAI, SLE disease activity index 2000; ALCAM, activated leukocyte cell adhesion molecule; HPX, hemopexin; PRDX, peroxiredoxin.

To assess the potential of the urinary biomarkers used in this study to evaluate the treatment response, additional analyses were conducted to examine the association between changes in biomarker levels during follow-up assessments and disease activity. The results of the correlation analyses between changes in SLEDAI-2k scores at baseline and follow-up and the corresponding changes in each biomarker’s values are presented in **Figure 5**. The change in SLEDAI-2k scores exhibited a positive correlation with changes in ALCAM (r = 0.502, p < 0.001), HPX (r = 0.475, p < 0.001), and PRDX6 (r = 0.245, p = 0.026), indicating that as the SLEDAI-2k score decreased, the levels of each biomarker decreased. We also investigated the changes in UPCR and corresponding changes in each biomarker in patients with LN (**Supplementary Figure 1**). Changes in ALCAM (r = 0.356, p = 0.01) and HPX (r = 0.316, p = 0.025) levels positively correlated with changes in UPCR. However, the relationship between ΔPRDX6 and ΔUPCR was not statistically significant.

## 4 Discussion

While active efforts are underway to develop of biomarkers for the diagnosis and prognosis of SLE, a comprehensive solution has not yet been fully realized. The inherent complexity of this disease poses challenges to its practical implementation in clinical settings. Consequently, there is an urgent need to develop more precise and specific biomarkers to enhance the accuracy of diagnosis and predict the treatment outcomes in patients with SLE. To address these challenges, this study focused on validating the potential of urinary biomarkers for SLE, specifically ALCAM, HPX, and PRDX6.

Our study on the three urinary biomarkers is noteworthy, as it is the first analysis targeting Korean patients with SLE. While previous research primarily focused on patients with LN, our investigation demonstrated the diagnostic value not only in LN but also in SLE without nephritis. Among the three biomarkers, ALCAM exhibited superior diagnostic capability, with an AUC exceeding 0.8 in both Korean patients with SLE and LN, consistent with existing research findings (15–17). ALCAM plays a pivotal role in inflammatory responses by actively participating in T cell co-stimulation and recruiting activated monocytes and T cells. In the presence of renal damage, ALCAM leads to the release of inflammatory cytokines, prompting additional recruitment of immune cells, including T cells, monocytes, inflammatory dendritic cells, neutrophils, and B cells (18, 19). In the event of lupus inflammation, there is an increase in the release of inflammatory cytokines through this mechanism, accompanied by elevated excretion of ALCAM in urine. This is particularly pronounced in patients with LN. Furthermore, S100B, known for its danger-associated molecular pattern activity, has been implicated in ALCAM-related mechanisms that induce inflammation through NF-κB activation (20).

Limited studies have been conducted on HPX and PRDX6 in adult patients with SLE. Our study revealed significant discriminative abilities of both HPX and PRDX6 in patients with SLE and LN compared with healthy individuals. Similar findings were reported in a study involving a small cohort of Chinese patients with LN (21). While previous studies on HPX have predominantly focused on child-onset lupus nephritis, recognizing its diagnostic and prognostic biomarker capabilities, our results align with studies targeting both child and adolescent-onset lupus nephritis patients (22–24). This similarity is presumed to be due to age-related correlations in HPX reference levels (25). HPX, an acute-phase reactant primarily produced in the renal cortex in response to nephrotoxic attacks, functions as a protein-degrading enzyme that protects renal tubules from the toxicity of free heme radicals. An increase in HPX is considered proportional to the severity of nephritis and is closely correlated with glomerular leukocyte infiltration, subendothelial deposits, and interstitial inflammation (26). In SLE patients without renal inflammation, elevated urinary HPX levels may result from inflammation or stress exposure (27). Elevated urinary HPX excretion rates could also reflect higher plasma levels of HPX.

While PRDX6 exhibited lower diagnostic capabilities than ALCAM and HPX, higher levels of urinary PRDX6 still maintained sufficient value associated with the diagnosis of SLE or LN. PRDX6 plays a dual role in inflammation by acting as an activator of the inflammatory pathway through NADPH oxidase associated with phospholipase A2 and a protective mechanism through its peroxidase activity (28). Conflicting results have been reported in studies targeting SLE, indicating the dual role of PRDX6 in inflammation. In this study, an increase in PRDX6 was observed in SLE and LN, suggesting a predominant inflammatory response through the elevated oxidative stress-mediated signaling pathway, which is consistent with findings in some autoimmune diseases (21, 29, 30). Additionally, patients with SLE demonstrate increased levels of PRDX6 protein expression compared with healthy individuals (31). Conversely, the analysis suggests that PRDX6 deficiency in B cells may upregulate mitochondrial respiration and antibody production, indicating a potential protective role for PRDX6 against organ damage in SLE (32). Further research is required to understand the mechanisms controlling the interplay between PRDX6 peroxidase and phospholipase A2 activity under pathological conditions.

Unfortunately, beyond LN, the three biomarkers did not demonstrate an association with clinical manifestations. Although ALCAM and HPX appear to be associated with CNS involvement in SLE, the limitation lies in the small number of patients with CNS involvement. The association between CNS involvement and urinary biomarkers is more likely to be attributed to an overall increase in lupus disease activity in patients with CNS involvement rather than a direct impact on the levels of CNS-involved biomarkers. Further investigation into the correlation between these biomarkers and CNS involvement may benefit from additional analysis of their levels in both the serum and cerebrospinal fluid (33).

In the present study, a significant finding is the strong association observed between the levels of ALCAM, HPX, and PRDX6 and disease activity. Notably, we emphasized the potential of these biomarkers as indicators for tracking disease activity over time. The baseline values of each biomarker showed a significant positive correlation with the SLEDAI-2k score, which is a popular tool for assessing SLE disease activity. These findings align with previous works (15–17, 21). However, a detailed comparative analysis of follow-up test results is lacking, with only one study reporting that an HPX-inclusive biomarker score could represent the response to induction therapy in patients with LN (34). Our study revealed a positive correlation between changes in biomarker levels and SLEDAI-2k scores, suggesting a highly promising outcome. In particular, the noninvasive measurement of these biomarkers in urine makes them preferable for frequent monitoring. The results of this study suggest that ALCAM, HPX, and PRDX6 have the potential to replace SLEDAI, offering practical and valuable indicators for real-time monitoring of disease progression or treatment effects without the need for cumbersome tests such as urinalysis for hematuria, urinary leukocytes, and cellular casts. Moreover, this study presents an avenue for evaluating the overall disease activity not only in patients with LN but also in the broader lupus population through urine analysis.

In summary, this study highlights the significance of urine ALCAM, HPX, and PRDX6 levels as diagnostic and therapeutic monitoring tools in a Korean population with SLE. This study introduces a safe and convenient method for evaluating disease activity in SLE. Especially, with the combination, the diagnostic ability is further enhanced, indicating that the diagnostic value of conducting all three tests is remarkably high. Considering all three biomarkers together allows for a more precise diagnosis of the disease, leading to the provision of appropriate treatment and management. However, this study has some limitations. First, the inclusion of samples from non-newly diagnosed patients may have influenced urinary biomarker concentrations, potentially because of concomitant medications such as immunosuppressants. Secondly, we were unable to analyze the correlation between renal tissue parameters such as LN classification or the active/chronic index and biomarker levels. Since LN patients exhibited higher anti-dsDNA levels, lower complements, and higher SLEDAI scores indicative of overall disease activity, it remains unclear whether LN manifestation and renal damage directly influence biomarker levels. Thirdly, variations in the timing of follow-up for urinary biomarker assessments among patients, determined by differences in outpatient visit schedules, pose a challenge. Cohort follow-up losses further limited the tracking of biomarker levels in all patients. In addition, practical challenges in simultaneously collecting urine samples in clinical settings impede the consideration of diurnal variations in urinary biomarkers. Finally, since HCs did not undergo other tests typically used to monitor disease activity in SLE, we couldn’t directly compare biomarker capabilities with commonly used indicators like C3, C4, and anti dsDNA. To evaluate the long-term clinical utility of urinary ALCAM, HPX, and PRDX6, it is crucial to address these limitations, validate the results across diverse patient groups, and conduct additional studies to establish their specificity as biomarkers for SLE.

## Data availability statement

The raw data supporting the conclusions of this article will be made available by the authors, without undue reservation.

## Ethics statement

The studies involving humans were approved by Ajou University Hospital Institutional Review Board. The studies were conducted in accordance with the local legislation and institutional requirements. The participants provided their written informed consent to participate in this study.

## Author contributions

J-WK: Investigation, Writing – original draft, Writing – review & editing, Data curation, Formal analysis. W-YB: Data curation, Formal analysis, Investigation, Writing – original draft, Writing – review & editing, Methodology. J-YJ: Formal analysis, Investigation, Writing – review & editing. H-AK: Formal analysis, Investigation, Writing – review & editing. S-WL: Writing – review & editing, Data curation, Methodology. C-HS: Writing – review & editing, Conceptualization, Funding acquisition, Investigation, Supervision, Writing – original draft.
