# Supplementary material for: Longitudinal assessment of urinary ALCAM, HPX, and PRDX6 in Korean patients with systemic lupus erythematosus: implications for disease activity monitoring and treatment response
Source: Front Immunol. 2024 Jun 10;15:1369385. doi: 10.3389/fimmu.2024.1369385 (PMC11194348; doi:10.3389/fimmu.2024.1369385)

Supplementary Materials

Supplementary Table 1. Results of renal biopsy classification in patients with LN

Renal histology (ISN/RPS classification), no. (%)	Patients with LN (N = 71)
Class I	2 (2.8)
Class II	3 (4.2)
Class III	10 (14.1)
Class IV	27 (38.0)
Class V	13 (18.3)
Class II+V	1 (1.4)
Class III+V	7 (9.9)
Class IV+V	8 (11.3)

ISN/RPS, International Society of Nephrology/Renal Pathology Section; LN, lupus nephritis.

Supplementary Table 2. Utility of ALCAM, HPX, and PRDX in urine for diagnosing SLE

Variable	AUC	CI	P value	Cut-off	Sensitivity	Specificity	PPV	NPV
ALCAM (pg/ml)	0.801	0.734 – 0.868	<0.001	1898.7	56.9	97.4	98.7	39.1
HPX (ng/ml)	0.707	0.622 – 0.792	<0.001	209.3	62.3	74.4	89.2	36.3
PRDX6 (ng/ml)	0.697	0.612 – 0.782	<0.001	0.2	58.5	82.1	91.9	36.4

ALCAM, activated leukocyte cell adhesion molecule; HPX, hemopexin; PRDX, peroxiredoxin; SLE, systemic lupus erythematosus; AUC, area under the curve; CI, confidence interval, PPV, positive predictive value; NPV, negative predictive value.

Supplementary Table 3. Utility of ALCAM, HPX, and PRDX in urine for diagnosing lupus nephritis

Variable	AUC	CI	P value	Cut-off	Sensitivity	Specificity	PPV	NPV
ALCAM (pg/ml)	0.850	0.778 – 0.921	<0.001	1935	71.6	97.4	98	65.5
HPX (ng/ml)	0.781	0.695 – 0.867	<0.001	212.5	73.1	74.4	83.3	60.4
PRDX6 (ng/ml)	0.714	0.617 – 0.812	<0.001	0.2	59.7	82.1	86	54.2

ALCAM, activated leukocyte cell adhesion molecule; HPX, hemopexin; PRDX, peroxiredoxin; AUC, area under the curve; CI, confidence interval, PPV, positive predictive value; NPV, negative predictive value.

Supplementary Table 4. Combination of three biomarkers in urine for diagnosing SLE

Variable	Positivity rate	AUC	CI	P value	Sensitivity	Specificity	PPV	NPV
ALCAM + HPX	73%	0.804	0.738–0.870	<0.001	56.8	97.4	81.0	44.4
ALCAM + PRDX6	96%	0.857	0.800–0.914	<0.001	72.3	92.3	85.1	56.3
HPX + PRDX6	88%	0.771	0.697–0.846	<0.001	59.1	89.7	76.9	50.0
ALCAM + HPX + PRDX6	97%	0.858	0.801–0.915	<0.001	72.4	92.3	84.9	60.0

ALCAM, activated leukocyte cell adhesion molecule; HPX, hemopexin; PRDX, peroxiredoxin; AUC, area under the curve; CI, confidence interval, PPV, positive predictive value; NPV, negative predictive value.

Supplementary Table 5. Combination of three biomarkers in urine for diagnosing lupus nephritis

Variable	Positivity rate	AUC	CI	P value	Sensitivity	Specificity	PPV	NPV
ALCAM + HPX	85%	0.856	0.786–0.927	<0.001	72.1	97.4	88.3	68.1
ALCAM + PRDX6	94%	0.884	0.821–0.948	<0.001	81.2	92.3	87.5	70.5
HPX + PRDX6	92%	0.829	0.752–0.906	<0.001	73.5	82.1	85.0	63.8
ALCAM + HPX + PRDX6	94%	0.888	0.824–0.953	<0.001	83.6	92.3	91.8	75.6

ALCAM, activated leukocyte cell adhesion molecule; HPX, hemopexin; PRDX, peroxiredoxin; AUC, area under the curve; CI, confidence interval, PPV, positive predictive value; NPV, negative predictive value.

Supplementary Figure 1. Correlation scatterplot of proteinuria and urine biomarker changes in follow-up evaluation of patients with lupus nephritis. (A) The relationship between Δ ALCAM and Δ UPCR. (B) The relationship between Δ HPX and Δ UPCR. (C) The relationship between Δ PRDX6 and Δ UPCR.

ALCAM, activated leukocyte cell adhesion molecule; UPCR, urine protein creatinine ratio; HPX, hemopexin; PRDX, peroxiredoxin.

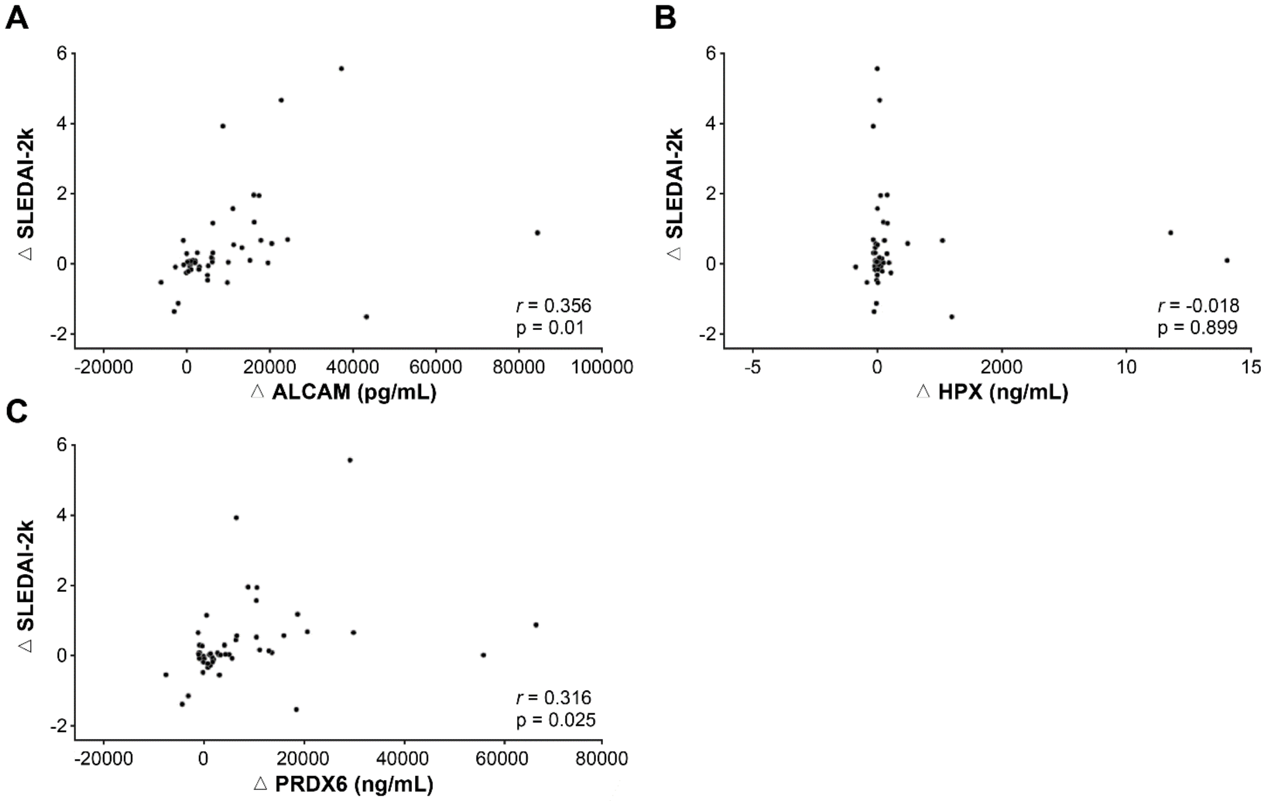

Supplementary Figure 2. Changes in urine ALCAM, HPX, and PRDX6 normalized for urine creatinine of patients with SLE without LN and patients with LN only follow-up. (A) Urine ALCAM/creatinine ratio. (B) Urine HPX/creatinine ratio. (C) Urine PRDX6/creatinine ratio. Statistical analyses were conducted using the Mann–Whitney U test. SLE, systemic lupus erythematosus; LN, lupus nephritis; ALCAM, activated leukocyte cell adhesion molecule; HPX, hemopexin; PRDX, peroxiredoxin.

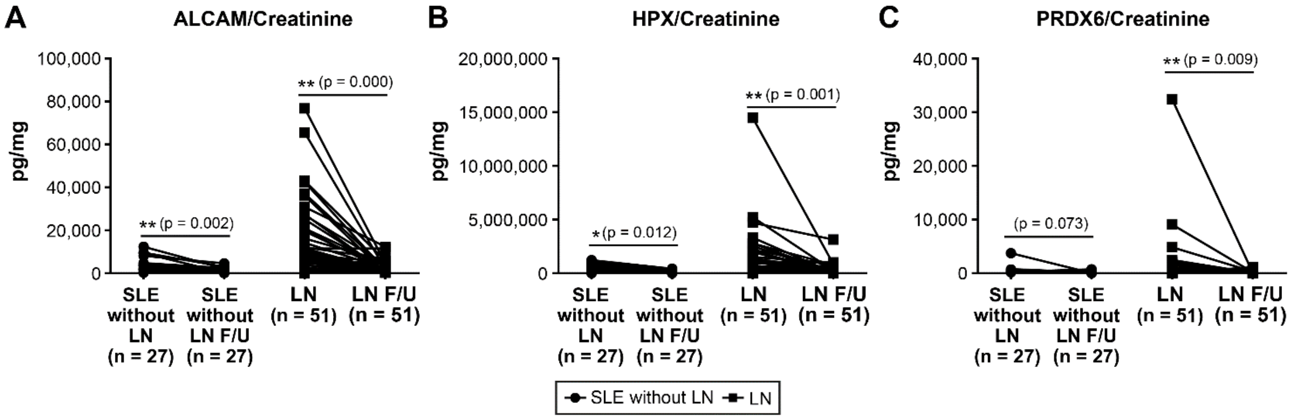

Supplement: Supplementary file 1 [file DataSheet_1.pdf]
